# Uncoupling of Carbonic Anhydrase from Na-H exchanger-1 in Experimental Colitis: A Possible Mechanistic Link with Na-H Exchanger

**DOI:** 10.3390/biom9110700

**Published:** 2019-11-05

**Authors:** Islam Khan, Khalid Khan

**Affiliations:** 1 Department of Biochemistry, Faculty of Medicine, Kuwait University, Jabriya 24923, Kuwait; 2 Department of Anatomy, Faculty of Medicine, Kuwait University, Jabriya 24923, Kuwait; Khalidkhanq8@gmail.com

**Keywords:** carbonic anhydrase, Na–H exchanger-1, myeloperoxidase, IBD, colitis

## Abstract

In this study, we investigated a mechanistic link between Na–H exchanger-1 (NHE-1) and carbonic anhydrase (CA) in experimental colitis induced in the rats by intrarectal administration of trinitrobenzenesulphonic acid (TNBS). Western blot analysis showed CA-I and CA-II as the major isoforms and CA-IV as a minor one in the colon, and they all are expressed as minor isoforms in the ileum. Co-immunoprecipitation and confocal immunofluorescence microscopy showed colocalization of NHE-1 with CA-I and CA-II, but not with CA-IV. TNBS significantly reduced the levels of NHE-1 and CA protein isoforms in the colon, but not in the uninflamed ileum. A similar reduction profile of the expression of CA isozymes was also obtained in ex vivo treatment of normal colon strips with TNF-α. The level of uncoupling as detected by co-immunoprecipitation was significantly more pronounced. A peptide (83 aa) from the NHE-1 C-terminus demonstrated binding of CA-II only, but not of the CA-I or CA-IV isoform. Furthermore, the profile of inflammatory test markers confirmed inflammation in the tissue used. These findings taken together suggest an inflammation-induced uncoupling of CA and NHE-1, which might be a putative mechanism for reducing the activity of NHE-1 in experimental colitis. This uncoupling might lead to an intracellular accumulation of H^+,^ resulting in acidosis and necrosis in the inflamed colon.

## 1. Introduction

Carbonic anhydrase (CA), a Zn metalloenzyme, catalyzes a reversible conversion of CO_2_ to HCO_3_^−^ and H^+^ under normal physiological conditions, and hence participates in the regulation of intracellular pH, and electroneutral uptake of NaCl and water from the renal tubules and gastrointestinal (GI) tract [1,2]. There are multiple isoforms of CA, which show a tissue-selective, and differential subcellular expression [3,4,5,6,7,8,9]. In the colonocytes, the cytoplasmic isozymes, CA-I and CA-II, produce H^+^ and HCO_3_^−^ from CO_2_, which are expelled out by NHE-1 and Cl^−^/HCO_3_^-^ transporters, respectively [8,9,10]. On the contrary, the CA-IV isozyme, present on the apical membrane in colonocytes, converts HCO_3_^−^ to CO_2_, which on percolation through the plasma membrane may fuel the cytoplasmic CA isozymes. A compromise, therefore, in the expression of these isozymes is likely to impair ion transport in inflammatory bowel diseases (IBDs). Crohn’s disease and ulcerative colitis represent IBDs, which are chronic inflammatory and debilitating conditions of the GI tract. Currently, the pathogenesis of IBD is believed to be a consequence of an exaggerated immune response to normal GI microflora and food antigens [11,12,13]. IBD conditions are frequently associated with diarrhea, pain, and altered muscle contractility. With regard to the role of other transporters in relation to Na–H exchanger-1 (NHE-1), our laboratory and others have reported impairment of Na^+^ transporting mechanisms including Na/K-ATPase (sodium pump) and Na–H exchanger (NHE) in the inflamed GI tract [14,15,16,17]. These changes may lead to increased amounts of Na^+^, Cl^−^, and water in the luminal contents. These findings, together with decreased amounts of HCO_3_^−^, K^+^, and butyrate in the luminal contents in IBD conditions, support our findings [18,19,20,21]. 

NHE-1 is fueled by the Na^+^ gradient, which is maintained by the sodium pump, and H^+^ concentration produced by the cytoplasmic CA. Overexpression of the CA-I isoform in transgenic mice has been implicated in chronic inflammation in arthritis [22]. On the contrary, decreases in the CA activity in ulcerative colitis, a chronic inflammatory, and other conditions have been also reported [10,23,24,25]. NHE-1 is regulated by several mechanisms, including a direct interaction of CA with the NHE-1 C-terminal segment [26]. Such interaction of CA with other transporters, such as anion exchanger (Cl^−^/HCO_3_^−^), is suggested to regulate the anion exchanger activity as well [27].

Therefore, an overall objective of this study was to investigate the mechanism of regulation of NHE-1 and CA expression in experimental colitis. In this study, localization of CA isozymes and NHE-1 was investigated using NHE-1-specific antibodies [28] by immunoprecipitation and confocal immunofluorescence microscopy techniques. Protein levels of CA-I, -II, -IV and NHE-1 isoforms, and their coupling were reduced in the inflamed colonic tissues. Ex vivo treatment of colonic strips with TNF-α also produced a similar reduction profile ascertaining a role of inflammation. The binding site of CA-II was mapped using an 83-aa peptide corresponding to the NHE-1 C-terminus [28]. In this study, we used the day 6 post-trinitrobenzenesulphonic acid (TNBS)-induced colitis, which has earlier been shown as inflamed in this laboratory.

## 2. Materials and Methods

Male Sprague Dawley rats weighing 200–250 gm used in this study were maintained by the Animal Facility, Health Sciences Center, Kuwait University in an air-conditioned environment with a dark and light cycle of 12 h each. Animals were provided with free access to water and normal feed, and handled following the standard ethical guidelines issued by the Animal Facility, Health Sciences Center, Kuwait University, Kuwait.

### 2.1. Induction of Colitis

Colitis was induced in the overnight fasted animals by delivering 30 mg TNBS (Fluka) dissolved in a 50% ethanol–phosphate-buffered saline (PBS) solution intra-rectally 8 cm from the anal margin with a syringe as described earlier [14,15,29]. The animals receiving PBS served as noncolitis controls. On day 6 post-TNBS, animals were sacrificed by cervical dislocation, and colonic and ileal segments were collected. Body weight recorded just before the induction of colitis was considered as the day 0 weight, and the weight recorded just before sacrifice was designated as the day 6 post-TNBS weight. Changes in the body weight were calculated and compared to their respective weights on day 0 [17,29]. Colon lengths and weights were also recorded on day 6, and changes were calculated with respect to the uninflamed colons in the controls. All protocols and methodology used in the present animal study followed the guidelines of the ethics committee on animal research of the Kuwait University with an approval number of MB 03/15. The Kuwait University follows the guidelines of animal handling in compliance with the International Council for Laboratory Animal Sciences.

### 2.2. Characterization of Colitis

Colitis was characterized by measuring myeloperoxidase (MPO) activity in the colonic segments, body weight, colon hypertrophy (weight per unit colon length), and light microscopic studies using H&E and alcian blue staining. MPO activity was measured using a standard colorimetric procedure and expressed as units per mg of tissue [28,29,30]. The enzyme unit is defined as the amount of the enzyme that catalyzes the conversion of one micromole of substrate per minute under the specified conditions of the assay.

### 2.3. Western Blot Analysis

#### 2.3.1. Preparation of Tissue Lysates and Crude Microsomes

Tissues were finely chopped with scissors and polytroned using ice-cold 4-morpholinepropanesulfonic acid (MOPS) buffer (pH 7.5). The lysates obtained were centrifuged at 5000× *g* (Sorvall) for 10 min [14,15,28,29], and supernatants collected after passing through a cheese cloth were further centrifuged at 10,000× *g* for 10 min. The levels of CA-I and CA-II proteins were measured using the filtrates. A portion of each supernatant was further centrifuged at 45,000× *g* for 45 min to obtain crude microsomes for measuring the levels of the membrane-bound CA-IV isozyme. All the steps were performed at 4 °C.

#### 2.3.2. Protein Concentration

Total protein concentrations in the lysates and crude microsomes were measured using a protein dye-binding assay kit (BioRad). The samples (1–3 mg/mL proteins) were prepared for gel electrophoresis using a standard sample buffer [28,29]. The samples were heated for 5 min in a boiling water bath just before loading onto a polyacrylamide gel.

#### 2.3.3. Protein Separation

Proteins were separated using a 12% polyacrylamide denaturing gel, and blotted onto a nitrocellulose or PVDF membrane electrophoretically overnight at 4 °C [14,15,28,31]. The membranes after blocking with a 5% nonfat milk solution in PBS for 30 min were incubated with 1° antibodies (1:2000 dilutions) separately in plastic bags containing a 5% nonfat milk solution for 2 h. At the end of the incubation, the membranes were washed three times with PBS for 5 min each. Subsequently, after the incubation with an appropriate dilution (1:2000) of the 2° antibody–HRP conjugates (SantaCruz, Germany) for 2 h, the membranes were washed for 5 min three times with PBS. All incubations were performed at room temperature with gentle shaking. Finally, the membranes were treated with the ECL reagents 1 and 2 (Amersham, UK) for 1 min, and exposed to X-ray films (Kodak, New York, NY, USA) for suitable times to obtain appropriate signals. Band densities from the X-ray film were recorded using a genetic analyzer. The expression level was calculated as a ratio of each isoform to the actin levels.

### 2.4. Co-Immunoprecipitation

Colonic tissue lysates were solubilized using triton X-100 and incubated with the NHE-1-specific antibodies at a dilution of 1:100 [28]. Immune complexes were captured using Protein-G sepharose beads (Pharmacia, Stockholm, Sweden), washed with an appropriate buffer and solubilized with 1× sample buffer (loading buffer). The proteins were separated on an 8% polyacrylamide gel and then transferred to a nitrocellulose membrane electrophoretically. The membranes were stained with CA isoforms-selective antibodies by ECL western blot analysis.

### 2.5. Confocal Immunofluorescence Localization

Confocal immunofluorescence localization of CA isoforms with NHE-1 was investigated using paraffin sections from blocks prepared using paraformaldehyde-fixed colonic tissues. Thin tissue sections (thickness: 7 µm) were placed on glass slides, deparaffinized, dehydrated and rehydrated using graded ethanol solutions as described earlier [17]. Epitopes were unmasked by microwaving and nonspecific binding sites were blocked using a 1% BSA solution. Then, the sections were incubated with the anti-NHE-1-, CA-I-, and CA-II-selective antibodies (1:25 dilution) overnight. The sections were washed and incubated with suitable 2° Ab–rhodamine (CA-I and CA-II) and 2° Ab–FITC (NHE-1) conjugates. Sections were stained with DAPI and stored in a mounting solution, and signals were visualized under a confocal microscope (Zeiss, Jena, Germany) using an appropriate monochromatic wavelength. Tissue sections incubated with 2° Ab conjugates only were also run simultaneously as negative controls [17].

### 2.6. Effect of TNF-α on the Expression of CA Isoforms

Colonic segments from noncolitis animals were isolated, rinsed with cold DMEM media and incubated with TNF-α (20 ng/mL DMEM) for 10 h at 37 °C in a CO_2_ incubator. The tissue strips incubated in a similar manner but without TNF-α served as controls. At the end of incubation, tissue lysates were prepared and used to examine the levels of CA-I and CA-II expression.

### 2.7. CA-Binding Site in the NHE-1 C-Terminal Peptide (CP)

A GST-fusion protein containing the NHE-1 CP (83 aa) prepared as described earlier [28] was separated on a 12% polyacrylamide gel by electrophoresis and electroblotted onto a nitrocellulose filter paper. The filter paper was blocked with a 5% milk solution in PBS and then incubated with 45 µg of tissue lysates from the controls and inflamed colons separately for 4 h. After washing with PBS, the filters were incubated with CA-selective 1° antibodies overnight at 4 °C separately (SantaCruz). All the steps were performed with gentle shaking. The nitrocellulose filters were washed with PBS and incubated with an appropriate HRP–2° antibody conjugate for 2 h at room temperature. Bands were developed using ECL reagents 1 and 2 and data analyzed.

### 2.8. Statistical Analysis

Data are presented as mean ± SE of duplicate determinations (*n* = 12). A non-parametric, unpaired, two-tailed t-test was performed using EXCEL, and a *p*-value of <0.05 was considered to be statistically significant. Data were compared with their respective control values.

## 3. Results

### 3.1. Characterization of Colitis

**Figure 1:**
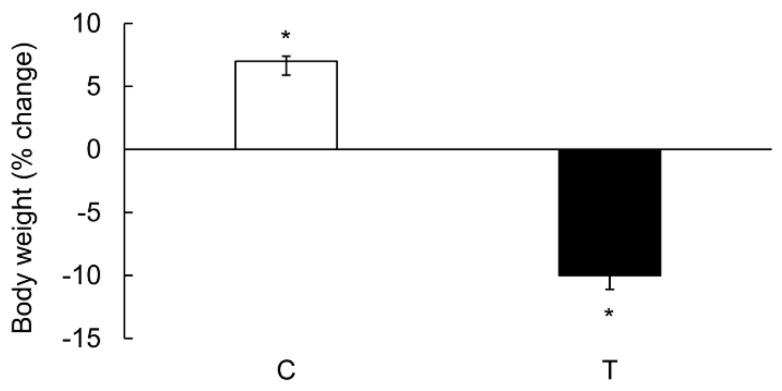
Bar diagram showing percent change in the body weight with respect to that on day 0 in the noncolitis controls (C, open bar) and in rats which received TNBS (T) 6 days earlier (closed bar). Data are presented as mean ± SE (*n* = 12). * indicates significance *p* < 0.05 with respect to their weight on day 0.

TNBS caused a significant reduction (*p* < 0.05) in the body weight in colitis animals as compared to their weight on day 0. The noncolitis control animals, on the contrary gained a significant body weight over the test period (Figure 1).

**Figure 2:**
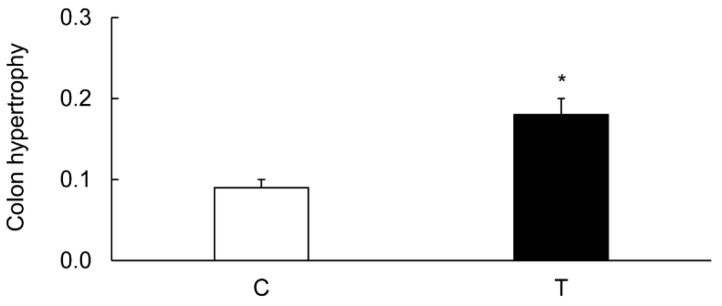
Bar diagram showing colon hypertrophy (g/cm colon) from the noncolitis controls (C, open bar) and the TNBS-inflamed colons (T, closed bar) from rats which received TNBS 6 days earlier. Data are presented as mean ± SE (*n* = 12). * indicates significance *p* <0.05 versus values for controls.

Weight and thickness of the inflamed colons were increased as compared to those of uninflamed colons. Colon hypertrophy represented as gm/cm colon length was also increased significantly in the TNBS-treated rats as compared to the noncolitis controls (Figure 2).

**Figure 3:**
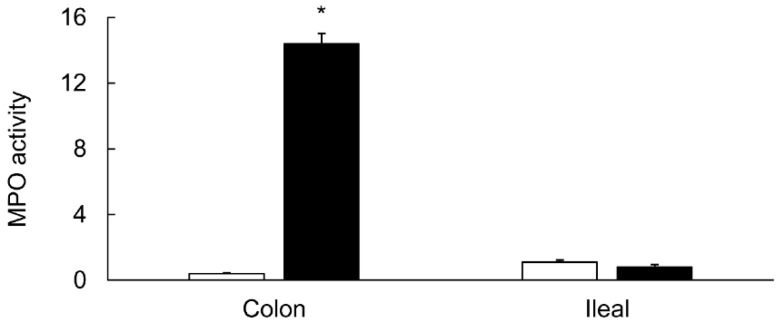
Diagram showing MPO activity as units/mg tissue in the indicated tissues from the noncolitis controls (open bars) and from animals which received TNBS 6 days earlier (closed bars). Data are presented as mean ± SE (*n* = 12). * indicates significance *p* <0.05 versus their respective control values.

MPO activity used as a biochemical marker for inflammation was also increased significantly in the inflamed colons as compared to the levels in the uninflamed colons (Figure 3).

**Figure 4:**
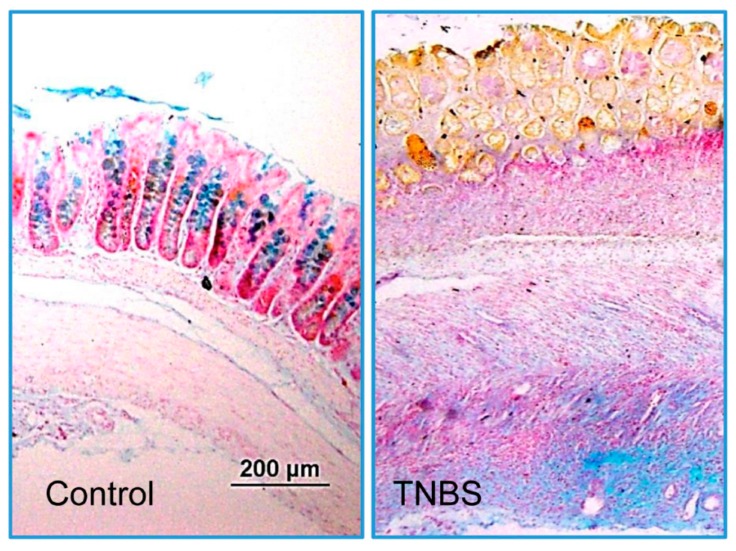
Representative light microscopic images showing H&E- and alcian blue-stained colonic tissue sections (5 μm) from the noncolitis controls and TNBS-treated rat colons. Magnification: 200×.

However, in the uninflamed ileum from the colitis animals, the level of MPO activity remained unchanged (Figure 3). Light microscopic studies showed an increased infiltration of inflammatory cells and a reduced intensity of alcian blue staining in the inflamed colons (Figure 4).

### 3.2. Protein Yield

There were no significant differences in the yield (mg/gm tissue) of total protein in the lysates or crude microsomal fractions from the inflamed and the uninflamed control colons (data not shown).

### 3.3. Expression of CA Isozymes

**Figure 5:**
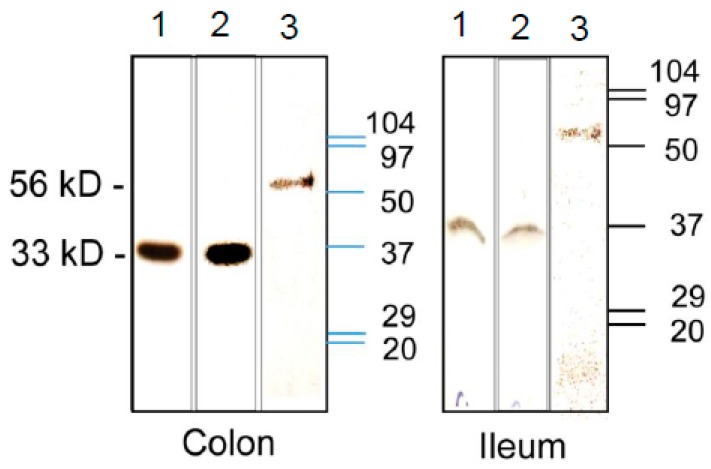
Representative (*n* = 3) ECL western blot analysis showing reactions of the carbonic anhydrase (CA)-selective antibodies with CA-I (lane 1), CA-II (lane 2), and CA-IV (lane 3) in the crude lysates (CA-I and CA-II) and microsomal fractions (CA-IV) from the colon (left panel) and the ileum (right panel). Low-range molecular weight protein size maker with band size are shown in kDa (Biorad).

The size of three CA reactive bands was confirmed separately before measuring their levels. The CA-I- and CA-II-selective antibodies reacted with a protein corresponding to a molecular mass of 29–35 kD, while the antibodies for CA-IV reacted with a 52–56 kD protein in both the colon and ileum (Figure 5). The CA-IV isozyme was detected in the crude microsomes, whereas the CA-I and CA-II isoforms were detected in the tissue lysates (Figure 5).

### 3.4. Colitis-Induced Expression of CA Isozyme 

**Figure 6:**
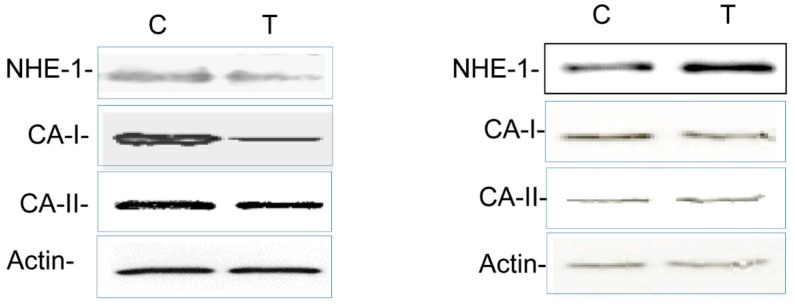
Representative ECL western blot analysis showing levels of the indicated protein isoforms in the noncolitis controls (C) and the TNBS-treated (T) colon (left panel) and ileum (right panel).

**Figure 7:**
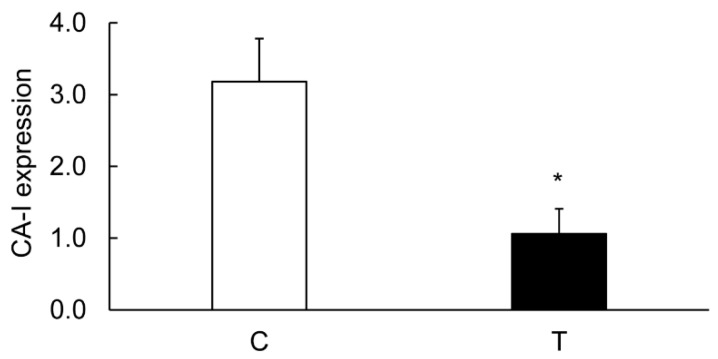
Bar diagram showing expression levels of CA-I in colon calculated as ratios with respect to actin as an internal control in the controls (C, open bar) and the TNBS-inflamed colon (T, closed bar). Data are presented as mean ± SE (*n* = 6). * indicates significance *p* of <0.05 with respect to the noncolitis controls.

**Figure 8:**
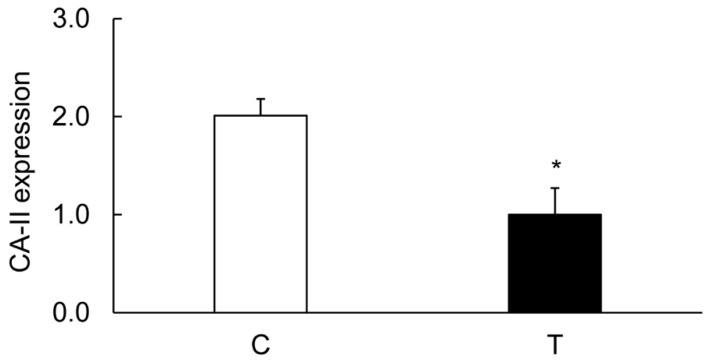
Bar diagram showing expression levels of CA-II in colon calculated as ratios with respect to actin as an internal control in the controls (C, open bar) and the TNBS-inflamed colon (T, closed bar). Data are presented as mean + SE (*n* = 6). * indicates significance *p* <0.05 with respect to the noncolitis controls.

**Figure 9:**
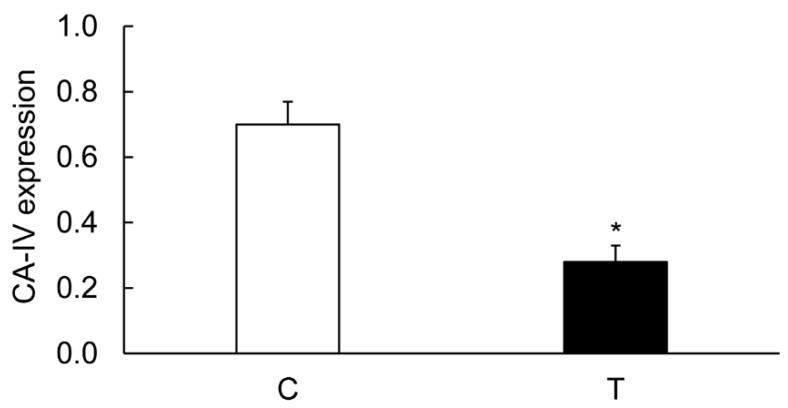
Bar diagram showing expression levels of CA-IV in colon calculated as ratios with respect to actin as an internal control in the controls (C, open bar) and the inflamed colon (T, closed bar). Data are presented as mean ± SE (*n* = 6). * indicates significance *p* <0.05 with respect to the noncolitis controls.

**Figure 10:**
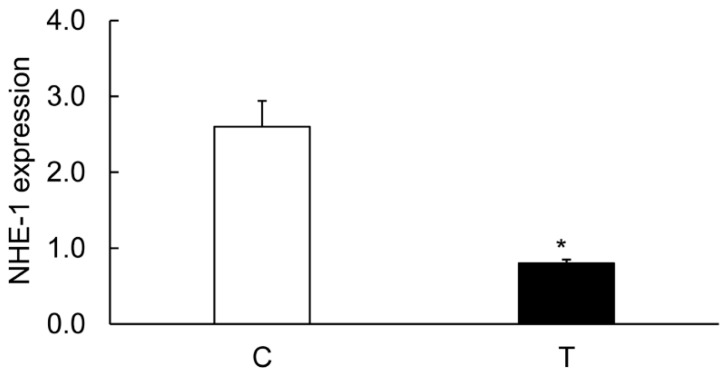
Bar diagram showing expression levels of NHE-1 protein calculated as ratios with respect to actin as an internal control in the controls (C, open bar) and the TNBS-inflamed colon (T, closed bar). Data are presented as mean ± SE (*n* = 6). * indicates significance *p* <0.05 with respect to the noncolitis controls.

The levels of CA-I (Figure 6 and Figure 7), CA-II (Figure 6 and Figure 8), and CA-IV (Figure 6 and Figure 9) isoforms were significantly decreased in the inflamed colon, whereas the level of actin remained unchanged. Similarly, the level of NHE-1 was decreased in the inflamed colons as compared to the noncolitis controls (Figure 6 and Figure 10).

**Figure 11:**
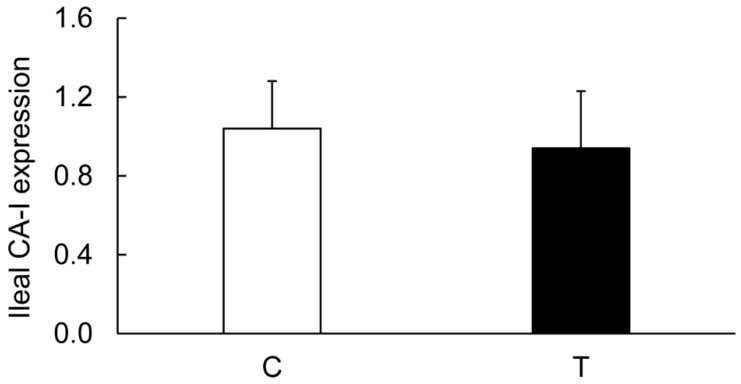
Bar diagram showing expression levels of CA-I in ileum calculated as ratios with respect to actin as an internal control in the ileum from the noncolitis (C, open bar) and TNBS-induced colitis (T, closed bar) animals. Data are presented as mean ± SE (*n* = 6).

**Figure 12:**
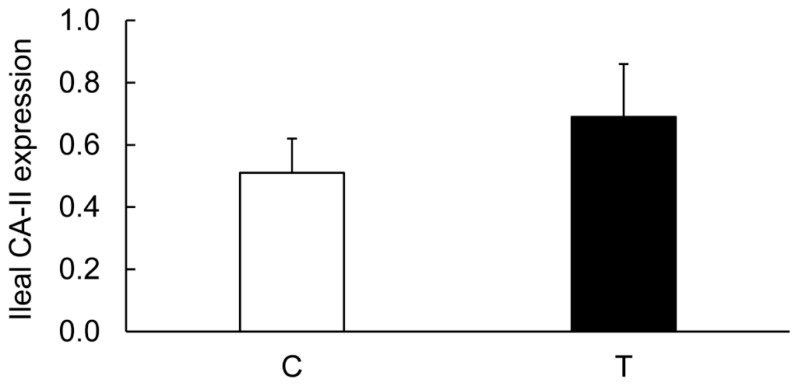
Bar diagram showing expression levels of CA-II calculated as ratios with respect to actin as an internal control in the ileum from the noncolitis (C, open bar) and TNBS-induced colitis (T, closed bar) animals. Data are presented as mean ± SE (*n* = 6).

**Figure 13:**
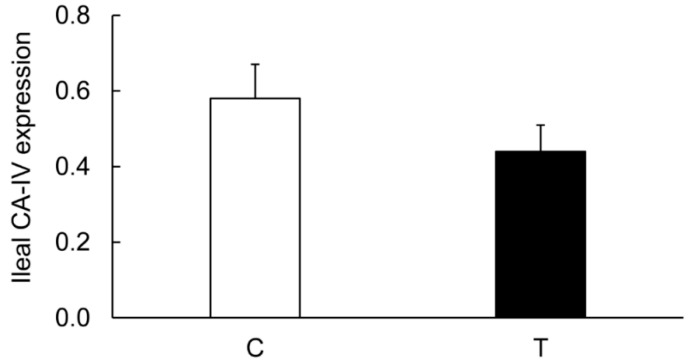
Bar diagram showing expression levels of CA-IV calculated as ratios with respect to actin as an internal control in the ileum from the noncolitis (C, open bar) and TNBS-induced colitis (T, closed bar) animals. Data are presented as mean ± SE (*n* = 6).

On the contrary, the levels of CA-I (Figure 6 and Figure 11), CA-II (Figure 6 and Figure 12), CA-IV (Figure 6 and Figure 13), NHE-1 isoforms, and actin in the ileum taken from the colitis animals remained unaltered.

### 3.5. Immunoprecipitation

**Figure 14:**
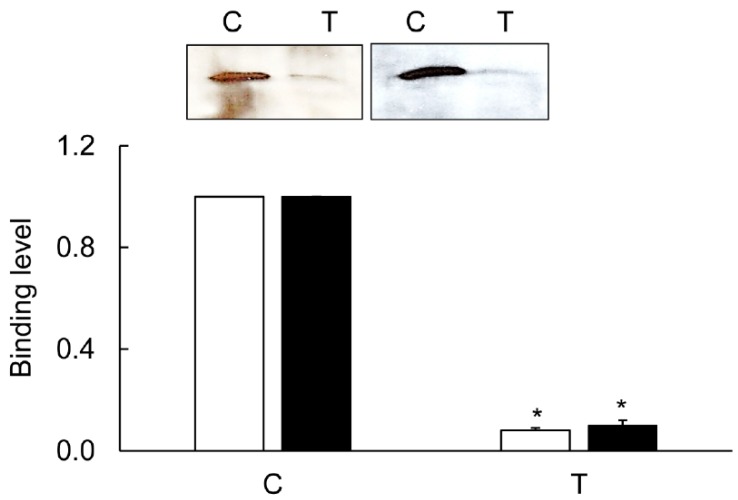
A representative western blot picture showing immunoprecipitation of CA-I (open bars) and CA-II (closed bars) binding levels with NHE-1 in the TNBS-inflamed (T) and uninflamed (C) colons. Inset: a representative western blot picture showing co-immunoprecipitation of CA-I (left panel) and CA-II (right panel) in the noninflamed controls (C) and TNBS-treated animals (T). Levels in the inflamed tissue are expressed relative to their respective control levels taken as 1.

An interaction between the CA isoforms and NHE-1 was examined in the material selected by the NHE-1-selective antibodies. The CA-I and CA-II isoforms, but not the CA-IV isoform, reacted with the NHE-1-pulled material (Figure 14). The level of CA isoforms was significantly less in the inflamed colon (Figure 14). The immunoprecipitation reduction in the CA-I and CA-II levels was >90%, while in the expression studies the reduction level was 40%–50% (Figure 7, Figure 8, and Figure 14).

**Figure 15:**
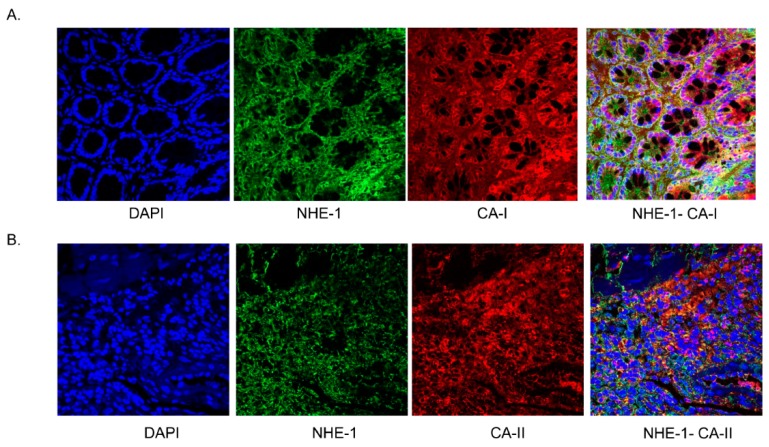
A representative picture showing confocal colocalization of CA-I (**A**) and CA-II (**B**) with NHE-1 in the noninflamed colonic segments. Colocalization is evident as yellow color signals in the superimposed picture (right most). Magnification: 20×.

Colocalization of CA-I and CA-II isoforms with NHE-1 was demonstrated by confocal microscopy (Figure 15A,B).

### 3.6. Mapping of the CA-Binding Site 

**Figure 16:**
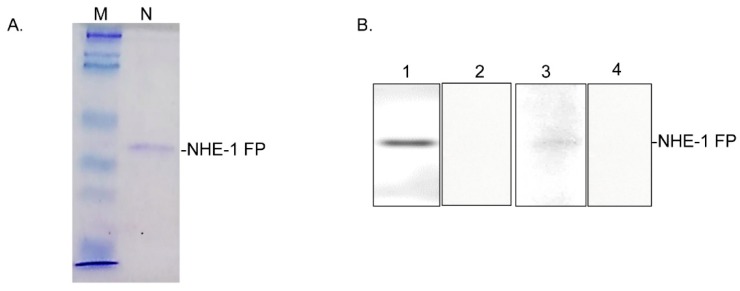
Representative pictures showing NHE-1 C-terminal peptide as a fusion protein on the polyacrylamide gel stained with coomassie blue dye (**A**), and its reaction with the anti-GST (lane 1), CA-I (lane 2), CA-II (lane 3), and CA-IV (lane 4) antibodies from the rat colon (**B**). M: Biorad protein marker, N: NHE-1 fusion protein (NHE-1 FP).

To further provide support for coupling of NHE-1 and CA isozymes and to map the binding site, we investigated whether there is an interaction between NHE-1 and CA isozymes with an NHE-1 FP (83 aa). The FP was prepared as a recombinant GST-fusion protein (Figure 16A) as described earlier [28]. The FP reacted with the CA-II isoform, but not with the CA-I or CA-IV isoforms (Figure 16B).

### 3.7. Effects of TNF-α on the Colonic Expression of CA-I And CA-II Ex Vivo

**Figure 17:**
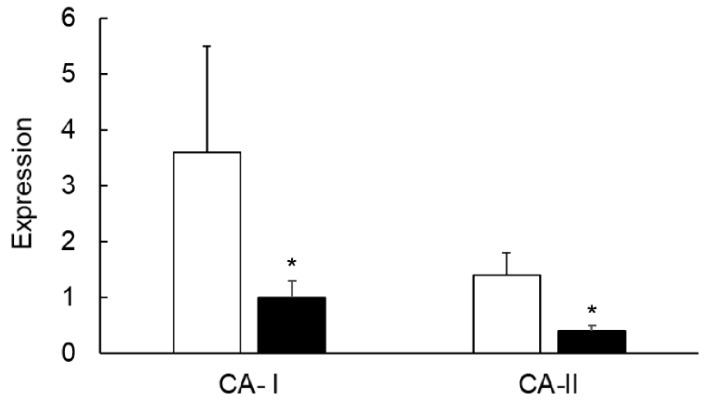
Bar diagram showing the effect of TNF-α on the expression of the indicated CA isozymes in the controls (open bars) and TNF-α-treated colonic strips (closed bars). Data are presented as mean ± SE (*n* = 5). * indicates significance *p* of <0.05.

TNF-α is a proinflammatory cytokine which is produced in inflamed colons. Blocking of this cytokine is routinely used as a treatment for Crohn’s disease. Our ex vivo experiments showed a significant suppression of both CA-I and CA-II in colonic strips treated with TNF-α, suggesting the role of inflammation in the regulation of CA expression (Figure 17).

## 4. Discussion

IBDs are associated with dysregulation of electrolyte and water homeostasis, which is attributed to an impairment of ion transport mechanisms. We have earlier reported a decrease in the expression of NHE-1 in inflamed colons in both the experimental colitis and human IBD conditions [14,15,29]. NHE-1 is a secondary active transporter regulated by a driving force of Na^+^ gradient and H^+^ concentrations, which are maintained by the sodium pump and CA, respectively. We and others have shown a down-regulation of sodium pump, which is suggested to suppress or down-regulate the NHE-1 activity in the inflamed colon [14,32]. Since protons are solely produced by CA, any decrease in its expression is likely to compromise the NHE-1 activity in the colitis as well. There is a preliminary report showing a decrease in the CA-I activity in ulcerative colitis due to the production of autoantibodies [25]; therefore, it is hard to predict whether this decrease in the CA-I activity is directly due to ulcerative colitis. In this study, we investigated roles of the CA isoforms in and their role in the down-regulation of NHE-1 in experimental colitis. Since both CA and NHE-1 work in close association with each other, it was important to first confirm their colocalization in the rat colon using immunoprecipitation and confocal immunofluorescence microscopy. Our hypothesis is that down-regulation of CA and/or its uncoupling from the NHE-1 is likely to affect the activity of NHE-1 in the inflamed colon. To study whether inflammation plays a role in the regulation of the CA expression, we used two approaches in this study: 1) we measured the levels of these protein isoforms in the noninflamed ileum from the colitis animals; 2) we extended our study using an ex vivo treatment of colonic strips with TNF-α, an inflammatory mediator. The experimental model used here has been very well studied for the investigation on the pathogenesis of IBD. In this study, we used the day 6 post-TNBS time point, which is known to exhibit inflammation. The discussion is focused on the alteration in the expression and colocalization of CA isoforms and NHE-1 in the inflamed colon, role of inflammation on the expression, uncoupling of NHE-1 and CA isoforms, and identification of the binding site of the CA isozymes in the NHE-1 C-terminus, and finally a physiological relevance of these findings is discussed.

The CA-I and CA-II isozymes are present in the cell cytoplasm, while the CA-IV isoform is present on the membrane surface facing the lumen. The cytoplasmic CA isozymes produce H^+^ and HCO_3_^−^. Protons thus produced serve as a fuel for the NHE-1 activity, whereas the HCO_3_^−^ is expelled out into the lumen by the Cl^−^/HCO_3_^−^ co-transporter. This, therefore, causes a net uptake of NaCl and water by the epithelial cells. The exterior CA-IV isozyme might produce CO_2_ from HCO_3_^−^ in the lumen, from where CO_2_ might enter the cell cytoplasm and complete the CA activity cycle. A decrease in CA proteins at two locations, therefore, may disrupt the cycle, and is likely to affect the activity of NHE-1. Our findings of reduced expression lead to an increased concentration of Na^+^, Cl^−^, and water in luminal contents, and provide support for the altered electrolyte contents reported earlier in diarrheal diseases [16,17,18,19]. In this study, we did not measure the NHE-1 or CA activity, and therefore, to further support our claim of activity, we measured alteration in the coupling of CA isoforms with NHE-1 using co-immunoprecipitation with an NHE-1 antibody-pulled-down material from the tissue lysates. Our findings demonstrated a reduction of >90% in the binding of CA-I and CA-II isoforms in the inflamed colon. The CA-IV isoform, however, did not show any coupling with the NHE-1 in the similar experimental conditions. Since NHE-1 activity is regulated by binding of CA in the NHE-1 C-terminus, these findings are interpreted as a reduction in the NHE-1 activity. Furthermore, a reduction in the expression of these proteins is specific as the protein yield, and actin levels remain unaltered in the inflamed colon. In addition, inflammation in the tissue used in this study was confirmed by changes in the body weight, colon hypertrophy, and MPO activity, consistent with our earlier findings in the same model. Furthermore, there was no change in the expression of these transporters in the uninflamed ileum, suggesting a role of inflammation in the reduced expression of CA isoforms. To further confirm the role of inflammation, we next examined the effects of TNF-α ex vivo on colonic strips. The levels of CA-I and CA-II were significantly decreased in the TNF-α-treated colonic tissues, suggesting that the reductions in the CA-I and CA-II were mediated by TNF-α.

A close association of NHE-1 and CA isoforms is important for supplying protons to NHE-1, so as to allow for their efficient expulsion from a cell and avoid cellular acidosis. This decrease in the expression as well as uncoupling increases the concentration of protons in the cell cytoplasm, leading possibly to acidosis and cell necrosis. For a neutral uptake of NaCl by NHE-1, an important role of Cl^−^/HCO_3_^−^ was predicted. There are multiple isoforms of the Cl^−^/HCO_3_^−^ exchanger [33], and therefore, a new project to systematically investigate their roles is required, which is a part of our ongoing studies.

In the inflamed colon, the decrease in the CA isoforms’ expression was approximately 40%–50% (Figure 7 and Figure 8); therefore other mechanism is likely to be instrumental in suppressing the activity of NHE-1. CA regulates NHE-1 activity by its direct binding to the NHE-1 C-terminal sequence [26,27]. To address this possibility, we examined the binding of the CA isoforms using crude tissue lysates with a C-terminal sequence of NHE-1 fusion protein prepared in this laboratory earlier [28]. Our findings from immunoprecipitation support binding of CA-I and –II isoforms, but not of the CA-IV isoform binding, with the NHE-1 isoform. This was further supported by confocal microscopy, showing colocalization of the CA-I and CA-II isoforms with NHE-1 in the rat colon. The binding of these two isoforms, CA-I and CA-II, was reduced by >90% (Figure 15) in the inflamed colon, which was significantly more than the reduction in the expression level. These findings, therefore, suggest an uncoupling of the CA as a possible mechanism for the reduction in the NHE-1 activity.

Next, we identified the binding sites of these isoforms using a GST-NHE-1 fusion protein containing an 83-aa peptide corresponding to the NHE-1 C-terminus [28]. Our findings showed the binding of CA-II isoform only, but not of the CA-I or CA-IV isoforms’ binding, with the NHE-1 fusion protein used in this study. Our findings support an earlier report showing an interaction of CA-II with an NHE-1 CP [26]. Since CA catalyzes hydration of CO_2_ to produce protons and bicarbonate, colocalization of CA with NHE-1 might be a mechanism to increase H^+^ availability to NHE-1 and thus improve an efficient removal of H^+^ from the cytoplasm without causing acidosis. Therefore, uncoupling of CA from NHE-1 is likely to impair NHE-1 activity in the inflamed colon, leading to accumulation of protons and thus acidosis and cell necrosis.

## 5. Conclusion

These findings taken together demonstrate an inflammation-induced suppression of CA isoforms in colons, which is mediated through TNF-α. Reduced expression, together with the uncoupling, accounts for the deregulation of NHE-1 expression in the inflamed colon. These changes compromise the uptake of NaCl and water from the lumen, leading to increased fecal concentrations of Na^+^, Cl^−^, and water, and decreased amounts of HCO_3_^−^ and butyrate uptake. It is worth noting that the short chain fatty acids, such as butyrate, are the chief source of energy for colonocytes [20,21]. Therefore, their uptake is likely to be suppressed due to suppression of CA-IV in this model.
